# Understanding the interplay between dNTP metabolism and genome stability in cancer

**DOI:** 10.1242/dmm.050775

**Published:** 2024-08-29

**Authors:** Miriam Yagüe-Capilla, Sean G. Rudd

**Affiliations:** Science For Life Laboratory (SciLifeLab), Department of Oncology-Pathology, Karolinska Institutet, 171 65 Stockholm, Sweden

**Keywords:** Deoxynucleoside triphosphate (dNTP) metabolism, Genome stability, DNA repair, Cancer

## Abstract

The size and composition of the intracellular DNA precursor pool is integral to the maintenance of genome stability, and this relationship is fundamental to our understanding of cancer. Key aspects of carcinogenesis, including elevated mutation rates and induction of certain types of DNA damage in cancer cells, can be linked to disturbances in deoxynucleoside triphosphate (dNTP) pools. Furthermore, our approaches to treat cancer heavily exploit the metabolic interplay between the DNA and the dNTP pool, with a long-standing example being the use of antimetabolite-based cancer therapies, and this strategy continues to show promise with the development of new targeted therapies. In this Review, we compile the current knowledge on both the causes and consequences of dNTP pool perturbations in cancer cells, together with their impact on genome stability. We outline several outstanding questions remaining in the field, such as the role of dNTP catabolism in genome stability and the consequences of dNTP pool expansion. Importantly, we detail how our mechanistic understanding of these processes can be utilised with the aim of providing better informed treatment options to patients with cancer.

## Teaser

**Summary:** This Review provides an overview of the current knowledge on both the causes and consequences of dNTP pool perturbations in cancer cells, together with their impact upon genome stability.

## Introduction

DNA is composed principally from the combination of four different deoxyribonucleoside triphosphate (dNTP) building blocks. These are classified according to their nucleobase moiety as purines (see Glossary, Box 1), comprising deoxyadenosine triphosphate (dATP) and deoxyguanosine triphosphate (dGTP), or pyrimidines (Box 1), comprising deoxycytidine triphosphate (dCTP), (deoxy)thymidine triphosphate (dTTP) and, lastly, deoxyuridine triphosphate (dUTP), which can be mis-incorporated by DNA polymerases instead of dTTP. As dNTPs are required for both the replication and repair of the nuclear and mitochondrial genomes, maintenance of dNTP homeostasis – a dNTP pool of the correct size and composition – is crucial for genomic integrity and cell fitness (Mathews, 2014). Considering this within the context of a cancer cell, which is subject to sustained proliferative signalling coupled with an inherently unstable and highly metabolised genome (Hanahan and Weinberg, 2011), this places dNTP supply in great demand. Accordingly, the relationship between dNTP pool size and composition and genome integrity is one that underpins several key aspects of oncogenesis, including elevated mutation rates and induction of certain DNA lesions (Box 1) (Mathews, 2015). Although therapeutic approaches aimed at dNTP metabolism are currently used in the clinic (Tsesmetzis et al., 2018), many open questions need addressing if we are to fully exploit this relationship to improve cancer treatment. In this Review, we compile the current knowledge on both the causes and consequences of dNTP pool perturbations in cancer cells, together with their impact upon genome stability. In doing so, we aim to outline several outstanding questions within the field and highlight how our mechanistic understanding of this interplay could be used to improve cancer treatment.

**Box 1. Glossary**

**Antimetabolite:** a widely used type of conventional chemotherapeutic agent that mimics endogenous metabolites (nucleobases, nucleosides or folate) and thereby interferes with metabolic processes in cells [e.g. deoxynucleoside triphosphate (dNTP) biosynthesis and nucleic acid metabolism].

**Copy number alterations:** deletions or amplifications of a particular DNA sequence within the genome.

**Chromosomal rearrangement:** a type of chromosomal abnormality involving a change in chromosome structure. For example, pieces of a chromosome could be missing (deletions), duplicated or moved around (inversions or translocations).

**DNA damage response (DDR):** a network of cellular processes that detect DNA lesions, signal their presence and initiate cellular responses, such as activation of cell cycle checkpoints and promoting repair.

**DNA lesions:** a broad term encompassing an array of chemical modifications and alterations to the structure of the DNA that can perturb normal DNA metabolism. Examples include single base modifications, base mismatches, bulky DNA adducts, inter-strand crosslinks, DNA-protein crosslinks, and single- and double-strand breaks.

**Genome instability:** a broad term referring to a high frequency of genetic alterations. This includes mutations; chromosome instability, in which there is a change in chromosome number; and microsatellite instability, in which repetitive DNA sequences in the genome are expanded or contracted owing to aberrant DNA replication and repair processes.

**Hypermutator phenotype:** a high frequency of point mutations within a cell. This can occur through loss of DNA replication fidelity-maintaining mechanisms, such as mismatch repair or DNA polymerase proofreading, together with alterations to dNTP pool composition.

**Nucleoside salvage:** A pathway that recovers bases and nucleosides from various sources (nucleic acids or exogenous sources) and converts them back into nucleotides. Of relevance to deoxynucleotides, this pathway consists of several deoxynucleoside kinases – deoxycytidine kinase (DCK), deoxyguanosine kinase (DGK), and thymidine kinase 1 and 2 (TK1 and TK2) – which generate deoxynucleoside monophosphates from deoxynucleosides.

**Oncogene-induced senescence (OIS):** an anti-proliferative response triggered by aberrant activation of oncogenic signalling within a cell.

**Purines and pyrimidines:** fundamental components of nucleotides; the building blocks of DNA and RNA. Purines (adenine and guanine) consist of two-ringed nitrogenous bases, whereas pyrimidines (cytosine, thymine and uracil) consist of a single-ring nitrogenous base.

**Replication fork:** a Y-shaped DNA structure in which the DNA duplex is unwound, producing two single-stranded templates to be replicated by DNA polymerases. Many proteins are located at replication forks to coordinate synthesis of the two DNA strands with other cellular processes.

**Replication fork collapse:** a consequence of stopped (stalled) replication forks that are not stabilised, or that persist for extended periods of time. The mechanisms, physical structure and protein composition of collapsed replication forks are an active area of investigation. Replication fork collapse can involve generation of a double-strand break.

**Replication stress:** a broad term referring to slowing or stopping (stalling) of replication fork progression and/or DNA synthesis. This could be caused by endogenous or exogenous processes. A unifying feature of replication stress is the generation of pathological excess single-stranded DNA. This can be generated when the DNA polymerase is slowed or stopped despite unwinding of the DNA duplex ahead proceeding.

**Ribonucleotide reductase (RNR):** the rate-limiting enzyme in the *de novo* production of dNTPs. RNR catalyses the reduction of ribonucleoside diphosphates to deoxyribonucleoside diphosphates, and is a long-standing anti-cancer target that can be inhibited by several chemotherapeutic agents, such as hydroxyurea and gemcitabine.

**Sterile α-motif and histidine-aspartic acid domain-containing protein 1 (SAMHD1):** a dNTP hydrolase that cleaves the triphosphate moiety from dNTP molecules. SAMHD1 is a key enzyme in dNTP catabolism and thus a major regulator of dNTP pools in cells, and also has non-catalytic functions in DNA repair.

## Multiple pathways control dNTP homeostasis

**Fig. 1.**
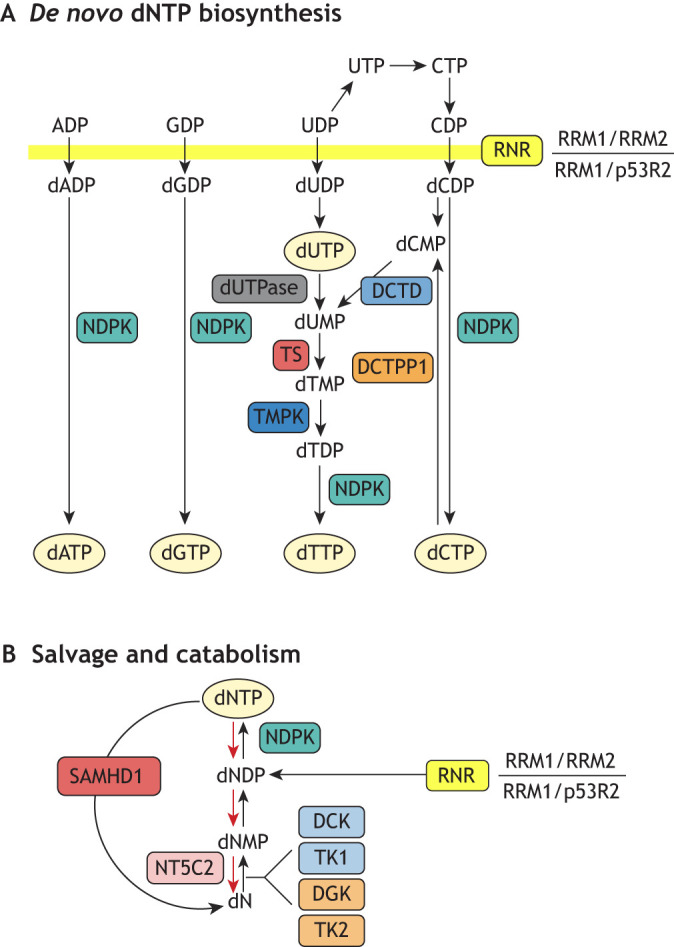
**Multiple pathways control dNTP homeostasis in cells.** The homeostasis of deoxynucleoside triphosphate (dNTP) pools depends upon the concerted action of *de novo* biosynthesis, nucleoside salvage and nucleotide catabolism (Mathews, 2014; Rampazzo et al., 2010). (A) The rate-limiting step in *de novo* biosynthesis is catalysed by ribonucleotide reductase (RNR), which can exist in oligomers consisting of a large RRM1 subunit and either small RRM2 or p53R2 subunits. Anabolism of deoxythymidine triphosphate (dTTP) also includes the action of enzymes responsible for deamination of deoxycytidine nucleotides (by deoxycytidine monophosphate deaminase or DCTD) or catabolism of deoxyuridine triphosphate (dUTP) (catabolised by dUTPase) to generate the thymidylate synthase (TS) substrate deoxyuridine monophosphate (dUMP). (B) The rate-limiting step in nucleoside salvage is catalysed by deoxynucleoside kinases – deoxycytidine kinase (DCK), deoxyguanosine kinase (DGK), and thymidine kinase 1 and 2 (TK1 and TK2) – which convert deoxynucleosides (dNs) into deoxynucleoside monophosphates (dNMPs). A principal factor in the catabolism of dNTPs is the dNTP triphosphohydrolase SAMHD1, which converts dNTPs back into their dN forms. Abbreviations: ADP, adenosine diphosphate; CDP, cytidine diphosphate; CTP, cytidine triphosphate; dADP, deoxyadenosine diphosphate; dATP, deoxyadenosine triphosphate; dCDP, deoxycytidine diphosphate; dCMP, deoxycytidine monophosphate; dCTP, deoxycytidine triphosphate; DCTPP1, deoxycytidine triphosphate hydrolase dCTPase; dGDP, deoxyguanosine diphosphate; dGTP, deoxyguanosine triphosphate; dNDP, deoxyribonucleoside diphosphate; dTDP, deoxythymidine diphosphate; dTMP, deoxythymidine monophosphate; dUDP, deoxyuridine diphosphate; GDP, guanosine diphosphate; NDPK, nucleotide diphosphate kinase; NT5C2, cytosolic 5′-nucleotidase II; TMPK, deoxythymidine monophosphate kinase; UDP, uridine diphosphate; UTP, uridine diphosphate.

In mammalian cells, the homeostasis of dNTP pools relies on multiple distinct pathways. These pathways have been expertly reviewed previously (Lane and Fan, 2015; Mathews, 2014) and are summarised in Fig. 1. In brief, three pathways contribute to the homeostasis of dNTP pools: (1) *de novo* biosynthesis, (2) nucleoside salvage (Box 1) and (3) nucleoside/nucleotide catabolism (Fig. 1). The *de novo* biosynthesis of dNTPs begins with the conversion of glucose to ribose 5-phosphate in the pentose phosphate pathway. Subsequently, this pathway diverges for the synthesis of purine and pyrimidine nucleosides before reconvening upon the rate-limiting enzyme in *de novo* dNTP production, namely, ribonucleotide reductase (RNR; Box 1). RNR is responsible for the reduction of ribonucleoside diphosphates (NDPs; specifically, those derived from adenosine, guanosine, cytidine and uridine) into their corresponding deoxyribonucleoside diphosphates (dNDPs), which can be subsequently phosphorylated to their triphosphate forms (dNTPs) (Nordlund and Reichard, 2006). A direct precursor of dTTP, however, is absent from the ribonucleoside pool and, thus, requires a different biosynthetic route using uracil-containing precursors. In this route, thymidylate synthase (TS, encoded by *TYMS*) produces the dTTP precursor deoxythymidine monophosphate (dTMP) from deoxyuridine monophosphate (dUMP). This can be generated either via deamination of deoxycytidine monophosphate (dCMP) by dCMP deaminase (DCTD) or following phosphorylation of RNR-generated deoxyuridine diphosphate (dUDP) to dUTP and subsequent hydrolysis by dUTPase (encoded by *DUT*) to generate dUMP (Reichard, 1988). The nucleoside salvage pathway operates in parallel to the *de novo* biosynthetic pathway and is controlled by a collection of deoxynucleoside kinases (Arner and Eriksson, 1995) – deoxycytidine kinase (DCK) and thymidine kinase 1 (TK1) in the nucleus, and deoxyguanosine kinase (DGK, also known as DGUOK) and thymidine kinase 2 (TK2) in the mitochondria – which phosphorylate existing deoxynucleosides into their monophosphate forms before additional nucleotide kinases generate the triphosphate metabolites. Opposing both these pathways is nucleotide catabolism, which allows control of dNTP pools through substrate cycles, converting dNTPs back to their monophosphate and deoxynucleoside forms (Rampazzo et al., 2010). This pathway is composed of nucleotidases, which remove single phosphates from monophosphate nucleotides (e.g. cytosolic 5′-nucleotidase II, NT5C2), together with the dNTP triphosphohydrolase sterile α-motif and histidine-aspartic acid domain-containing protein 1 (SAMHD1; Box 1) (Franzolin et al., 2013), capable of removing all three phosphates from a dNTP molecule (Goldstone et al., 2011; Powell et al., 2011). Altogether, these distinct pathways, governed in part by allosteric feedback control mechanisms, collaborate to maintain a dNTP pool of adequate size and composition throughout the cell cycle.

But what is a pool of adequate size and composition (other than one that allows a cell to function)? From experiments with cultured cells, dNTP pools typically range from 10 to 100 µM, with pool sizes increasing 5- to 10-fold when cells proliferate and duplicate their genome (Ferraro et al., 2010, 2005). With regards to composition, the dGTP pool is frequently observed as the smallest pool, followed by dCTP and dATP pools, with dTTP having the largest pool, which is approximately 5-fold larger than that of dGTP. Differences are also found between nuclear and mitochondrial relative ratios; in mitochondria, dGTP levels are expanded compared to the levels of other nucleotides. However, most mitochondrial dGTP is bound to the inner mitochondrial membrane (i.e. it is not available for DNA replication), highlighting that the technique used for the measurement of dNTPs is crucial and that the reported dNTP levels may not be completely associated to their roles in DNA metabolism (Molina-Granada et al., 2022). Thus, as variations in recorded dNTP levels and composition exist, and can depend upon many factors (e.g. cell or tissue type and method used), there are important efforts within the community to collate this information (Pancsa et al., 2022).

## Causes of dNTP pool perturbations in cancer cells

**Fig. 2.**
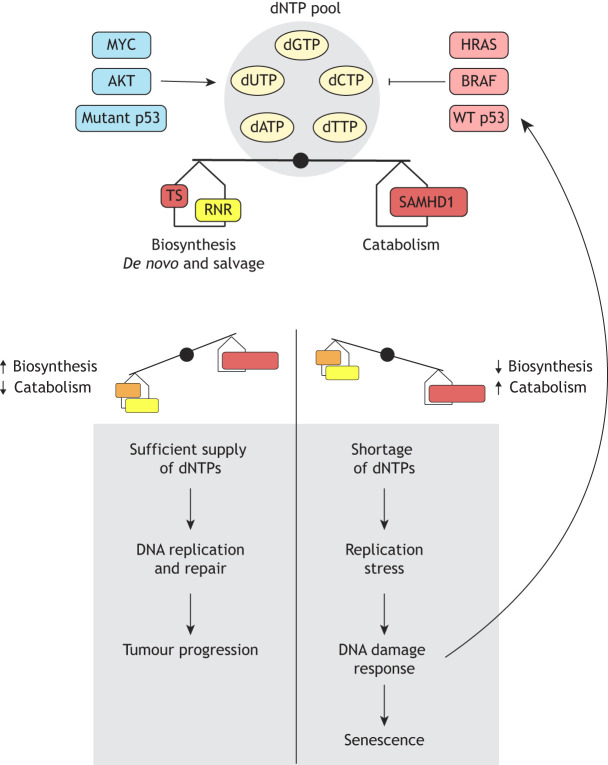
**Enzymes involved in dNTP metabolism are regulated by different genes to induce or interrupt tumour progression.** The levels of deoxynucleoside triphosphates (dNTPs) rely on the finetuned regulation of the enzymes involved in their biosynthesis, which comprises both the *de novo* pathway, the salvage of pre-existing nucleosides and their catabolism. Thus, if biosynthesis is promoted, the provision of dNTPs will support DNA replication and repair, required to sustain tumour growth. This situation can occur through the action of several oncogenes, such as *MYC* and AKT genes, or by the additional roles of mutant p53, due to upregulation of enzymes involved in dNTP production. In contrast, to avoid carcinogenesis, oncogene-induced senescence can occur, for example, as a result of expression of oncogenic HRAS and BRAF, which lead to suppression of the genes regulating dNTP production. As a consequence, shortage of dNTPs will lead to inadequate levels not compatible with DNA synthesis, triggering replication stress and the DNA damage response, which activates wild-type (WT) p53 and boosts this process, promoting the senescence of tumour cells. Abbreviations: dATP, deoxyadenosine triphosphate; dCTP, deoxycytidine triphosphate; dGTP, deoxyguanosine triphosphate; dTTP, deoxythymidine triphosphate; dUTP, deoxyuridine triphosphate; RNR, ribonucleotide reductase; TS, thymidylate synthase.

Despite the multi-layered system for ensuring dNTP pool homeostasis (Fig. 1), numerous factors can compromise this balance, leading to depletion, imbalance or expansion of dNTP pools (Fig. 2) (note that these are not mutually exclusive, as depleted or expanded pools can also be imbalanced). These factors include (dys)regulation of nucleotide metabolism by oncogenes and tumour suppressors, mutation or altered expression of the genes encoding nucleotide metabolic enzymes, or, in some cases, the induction of DNA damage, as discussed below. Additionally, perturbation of non-dNTP metabolic enzymes, such as those involved in DNA metabolism, can also impact dNTP pools, which has been discussed previously (Pai and Kearsey, 2017).

### Oncogenes and tumour suppressors

Oncogenes and tumour suppressors can directly control the expression of nucleotide metabolic enzymes. For instance, this is well documented in oncogene-induced senescence (OIS; Box 1), a process that entails cell cycle exit and subsequent growth arrest upon expression of an oncogene, which serves as a critical barrier to tumorigenesis. Multiple mechanisms drive OIS (Kotsantis et al., 2018), including downregulation of nucleotide metabolism (Aird and Zhang, 2015). This effectively starves proliferating cells of dNTP substrates required for DNA synthesis and thus promotes replication stress (Box 1), the slowing or stopping of DNA replication, and the uncoupling of leading and lagging strand DNA synthesis, leading to the generation of excessive single-stranded DNA (Kotsantis et al., 2018). This can be further compounded by increased firing of replication origins (the initiation sites of DNA synthesis), which is another consequence of oncogene induction that could increase the rate of dNTP consumption (Beck et al., 2012; Kotsantis et al., 2018).

The importance of dNTP supply is underscored by the finding that replication stress in cancer cells following oncogene induction can be rescued by supplementing the cell medium with nucleosides (Aird et al., 2013; Bester et al., 2011; Mannava et al., 2013; Maya-Mendoza et al., 2015) or folate (Lamm et al., 2015), a micronutrient required for dNTP biosynthesis. Replication stress and the induction of DNA damage activates the DNA damage response (DDR; Box 1) (Bartkova et al., 2006; Di Micco et al., 2006), largely controlled by the ataxia telangiectasia mutated (ATM) and ataxia telangiectasia and Rad3-related (ATR) kinases, which in turn activate the p53 (also known as TP53)–p21 (CDKN1A) and p16 (CDKN2A)–pRB (RB1) pathways to induce senescence (Serrano et al., 1997). In relation to dNTP metabolism, mechanistically, it was demonstrated that oncogenes can suppress expression of RRM2, the small subunit of RNR. This occurs via the replacement of its transcriptional activator E2F1 with the transcriptional repressor E2F7. Downregulation of RRM2 leads to depletion of dNTP pools and promotes replication stress, activating the DDR and in turn p53, which positively feeds back by further increasing E2F7 activity (Aird et al., 2013). This suppression of RRM2 expression was observed with both HRAS^G12V^- and BRAF^V600E^-mediated oncogene induction in human fibroblasts and melanocytes, respectively (Aird et al., 2013). HRAS^G12V^ expression in human fibroblasts was also found to suppress TS expression, crucial for dTTP biosynthesis, and expression of the large subunit of RNR, RRM1 (Mannava et al., 2013). However, tumour cells can evade OIS when one of these pathways is circumvented, alleviating replication stress and again promoting cell proliferation. In this situation, dNTP biosynthesis is indeed favoured to maintain cancer cell growth.

Notably, dNTP biosynthesis can be promoted by the inactivation of the p53 and LKB1 (also known as STK11) tumour suppressors, or by activation of MYC, RAS and AKT oncogenes (Fig. 2) (Villa et al., 2019). For example, MYC binds to the promoters and upregulates most components of purine and pyrimidine biosynthesis, which has been documented in several preclinical cancer models (Liu et al., 2008). Accordingly, MYC depletion negatively affects the expression of nucleotide biosynthetic enzymes such as TS, required for dTTP production, and inosine monophosphate dehydrogenase 2 (IMPDH2), which catalyses an important step in guanine nucleotide biosynthesis (Mannava et al., 2008). Melanoma cells depleted of MYC downregulate TS and RNR and accumulate DNA damage and senescence-associated phenotypes, which can be rescued by overexpression of these enzymes or exogenous treatment with deoxynucleosides (Mannava et al., 2012). Thus, although apparent MYC targets vary between studies, it is well evidenced that MYC promotes dNTP biosynthesis.

In contrast, tumour suppressors can halt tumour progression by limiting nucleotide provisions, generally achieved through downregulation of nucleotide biosynthetic enzymes. A notable example includes the widely studied effects of p53 on dNTP metabolism, where wild-type p53 is known to downregulate RNR (He et al., 2017). Several studies have shown that mutant p53 is associated with the activation of several enzymes involved in dNTP biosynthesis (including RRM2 and TS) and salvage (including DCK and TK1) (Kollareddy et al., 2015; Villa et al., 2019). Furthermore, metabolomic and transcriptomic studies of p53 have also revealed that most differentially expressed genes in the absence of p53 are involved in nucleotide and dNTP metabolism (Huang et al., 2018).

Altogether, it is becoming increasingly clear that the key architects of cancer biology, namely, oncogenes and tumour suppressors, achieve control of malignant biology in part by regulating dNTP metabolism, which in turn can be reinforced through positive feedback mechanisms through induction of DNA replication stress and activation of the DDR (Fig. 2). A key area of interest here is the role of dNTP catabolism, as much of our understanding to date has centred upon the control of biosynthesis, but dNTP abundance can also be controlled at the level of degradation.

### Perturbation of dNTP metabolic enzymes

Reflecting its central role in *de novo* dNTP biosynthesis, high expression of the small RNR subunit RRM2 has been reported in a variety of cancer types and correlates with worse prognosis (Liu et al., 2013, 2012; Morikawa et al., 2010a,b; Wang et al., 2012; Zhou et al., 2022). This is consistent with findings discussed earlier that elevated expression of RRM2 can overcome the tumour suppressive barrier of OIS (Aird et al., 2013; Mannava et al., 2013). Upregulation of RRM2 has also been linked to accumulation of genomic uracil [as RNR reduces uridine diphosphate (UDP) to dUDP, which can then be phosphorylated to the DNA polymerase substrate dUTP], particularly when dUTPase levels are low, resulting in replication stress and genome instability (Box 1) (Chen et al., 2016). Indeed, analysis of alterations in gene expression in different cancer types places RRM2 among the top 10% of the most upregulated genes in 73 of the 168 total cancer types studied (Aye et al., 2015). In agreement, a systematic meta-analysis of publicly available microarray data revealed that, among other nucleotide biosynthetic enzymes, RRM2 was upregulated in human tumours (Nilsson et al., 2014). It has also been suggested that the role of RRM2 in tumorigenesis goes beyond the mere expansion of dNTPs for sustained cell proliferation, as it has been reported that upregulation of RRM2 induces lung tumorigenesis in a mouse model through a mutagenic mechanism, with the most frequently mutated gene being the proto-oncogene *KRAS* (Xu et al., 2008). Upregulation of TS has also been associated with tumour transformation in mice, although the precise mechanism has not been deciphered (Rahman et al., 2004). However, in contrast, upregulation of RRM1 is correlated with suppression of tumour progression and lung metastasis (Fan et al., 1997), leading to the suggestion that it is the balance between these two subunits that controls tumorigenic processes.

As the control of dNTPs depends on the correct balance between synthesis and degradation, expansion of the pool of dNTPs available for genome duplication can also be achieved by impairing the activity of catabolic enzymes such as SAMHD1. Indeed, SAMHD1 is implicated in Sézary syndrome, a type of T-cell lymphoma, for which there is an inverse association between SAMHD1 expression and malignancy (de Silva et al., 2014). Additionally, recurrent *SAMHD1* mutations have been found in a variety of haematological malignancies, such as chronic lymphocytic leukaemia (CLL) (Clifford et al., 2014; Landau et al., 2013; Schuh et al., 2012), T-cell prolymphocytic leukaemia (Johansson et al., 2018) and mantle cell lymphoma (Buhler et al., 2021; Nadeu et al., 2020; Roider et al., 2021; Wang et al., 2021). These findings have also been corroborated by bioinformatic analyses of data derived from solid tumours, which highlight a mutational landscape of *SAMHD1*, with the most prevalent mutations in endometrial, thyroid, skin, colon and liver cancer (Schott et al., 2022). Mutations in *SAMHD1* in different cancer tissues have also been reported by the Catalogue of Somatic Mutations in Cancer (COSMIC) (Tate et al., 2019). A specific R366C/H SAMHD1 mutation has been found in leukaemia and colon cancers, which ablates the dNTP hydrolase activity of SAMHD1 while retaining catalytic-independent functions, and could thus mechanistically contribute to elevated dNTP pools in cancer cells (Bowen et al., 2021). Co-occurrence of *SAMHD1* mutations with mutations in genes encoding mismatch repair (MMR) components, a major pathway in maintaining DNA replication fidelity, has been observed in colon cancer (Rentoft et al., 2016). In the same study, the authors demonstrate that minor dNTP pool alterations can dramatically increase mutation rates in combination with MMR deficiency in yeast and human cancer cells (Rentoft et al., 2016), highlighting how the potential loss of SAMHD1 dNTPase activity and MMR could both contribute to the mutator phenotype of cancer cells. Besides, similarly to RNR, although the direct catalytic activity of SAMHD1 may be responsible for the impact of its expression on cancer development, its participation in additional processes, such as DNA damage repair (see Box 2), may also be important. Indeed, loss of the DNA repair functions of SAMHD1 are implicated in the progression of CLL (Clifford et al., 2014). Thus, in opposition to RNR subunit-encoding genes as putative oncogenes in malignant transformation and progression, SAMHD1 has been suggested as a tumour suppressor and mutations in *SAMHD1* have been proposed as drivers of oncogenesis, as not only is it important to maintain correct dNTP levels, but it also has a pivotal role in DNA repair and restart of stalled replication forks (Box 1) (Coquel et al., 2018; Daddacha et al., 2017; Kapoor-Vazirani et al., 2022; Liu et al., 2023).

**Box 2. Non-catalytic roles of dNTP metabolic enzymes in DNA repair**

In addition to enzymatic roles at DNA repair sites, there are examples of nucleotide and deoxynucleoside triphosphate (dNTP) metabolic enzymes with non-enzymatic roles in DNA repair. Thus, the localisation of metabolic enzymes at damaged sites is not necessarily confirmation of a metabolic role in the DNA repair process. For instance, the dNTP hydrolase SAMHD1 is recruited to DNA damage sites (Clifford et al., 2014), where it has non-catalytic roles in DNA break repair and replication fork restart via interaction with repair nucleases (Coquel et al., 2018; Daddacha et al., 2017). These functions could have potential implications in the response to DNA-damaging chemotherapeutic agents (Daddacha et al., 2017) and leukaemia development (Clifford et al., 2014). MTHFD2, an enzyme in mitochondrial folate one-carbon metabolism, required to fuel nucleotide biosynthesis, has an emerging non-catalytic role in nuclear DNA repair, promoting DNA break repair via interaction with DNA repair enzymes (Li et al., 2021; Yue et al., 2020).

Altogether, although SAMHD1 and RNR have been profoundly studied individually, as their complementary regulation and activity dictates dNTP homeostasis, further studies are required to better understand their specific interaction. Indeed, SAMHD1 deficiency could indirectly influence RNR, as changes in dNTP levels could allosterically regulate RNR and further impact on the specificity towards the dNTPs to be synthesised (Franzolin et al., 2020). In a similar fashion, RNR inhibition leads to apparent indirect inactivation of SAMHD1 drug resistance activity, probably owing to imbalances in dNTP levels and perturbation of SAMHD1 allosteric regulation, highlighting their tight interconnection, which can be exploited for cancer therapy (Rudd et al., 2020). When considering the mode of action of RNR inhibitors as monotherapies, an important point for future investigation is how SAMHD1 status can affect the efficacy of these therapies, given the central and opposing roles of SAMHD1 and RNR in dNTP homeostasis.

### DNA damage-induced changes in dNTP pools

It is not only dysregulation of specific genes in cancer cells that can influence dNTP pool composition, but also the treatments employed. In this context, one important class of chemotherapies in use is antimetabolites (Box 1), which exploit nucleotide metabolic pathways to target genomic integrity in cancer cells (Helleday and Rudd, 2022). In fact, many long-standing drugs used in oncology target the DNA, inducing distinct DNA lesions, and a key cytotoxic lesion is replication-dependent double-strand breaks (DSBs) (Helleday et al., 2008). As dNTPs are essential for DNA repair, a general assumption would be that dNTP biosynthesis should be promoted because of DNA damage. This phenomenon has been well documented in yeast, as it was observed in *Saccharomyces cerevisiae* and *Schizosaccharomyces pombe* that an acute increase in the dNTP pools upon DNA damage leads to increased survival, though in exchange for higher mutation rates (Chabes et al., 2003; Hakansson et al., 2006a). In human cells, the association of DDR to dNTP biosynthesis and its consequence is more complex than in single-celled organisms. It has been reported that ATM phosphorylates p53R2 (also known as RRM2B), the p53-inducible small subunit of RNR, and improves its stability (Chang et al., 2008). In a similar fashion, it has been observed that ATR signalling also inhibits RRM2 degradation (D'Angiolella et al., 2012). Both these strategies are aimed at extending the timespan of RRM2 activity after DNA damage. Furthermore, both wild-type p53 and some p53 mutants can induce the expression of RRM2 following DNA damage (Nakano et al., 2000; Schmidt et al., 2017; Tanaka et al., 2000). However, although RNR stability is induced upon DNA damage, expansions in dNTP pools were not observed in mammalian cells and no differences in the dNTP levels were detected after induction of DNA damage, presumably to avoid mutagenesis – which is more detrimental to multicellular organisms – that could arise from higher RNR expression (Das et al., 2022; Hakansson et al., 2006b). In agreement, treatment with several types of oncology drugs that cause DNA lesions and presumably trigger DDR led to either unperturbed dNTP levels or disturbances in dNTP ratios, though not a significant expansion of the dNTP pools. Only in the case of the antimetabolite gemcitabine was dTTP accumulation observed, explained by the upregulation of TS and TK1 (Huang et al., 2020). Similarly, a reduction of dATP, dGTP and dCTP levels was observed in cancer cell lines upon treatment with the oncology drugs 5-fluorouracil and methotrexate, whereas the nucleoside analogue cytarabine depleted dATP and dGTP (Liu et al., 2014). However, these drugs not only induce DNA damage, but can also directly perturb activities of dNTP metabolic enzymes. Other drugs were analysed that led to different nucleotide perturbations, but only the microtubule inhibitor vincristine showed expansion of dNTPs (specifically, dATP, dGTP and dCTP) (Liu et al., 2014). The accumulation of nucleotides has also been observed following irradiation, suggested as a consequence of the specific phosphorylation and activation of PRPS1/PRPS2 activity, mediated by ATM (Liu et al., 2021). However, these results have been questioned (Das et al., 2022), as the authors only studied the enzymes PRPS1/PRPS2 and no other downstream components of nucleotide and dNTP biosynthesis were included. Besides, it is inconsistent with RNR feedback inhibition by dATP, which would restrict the expansion of dNTPs (Das et al., 2022).

As recruitment of RNR to DNA lesions has been observed (Niida et al., 2010), an alternative hypothesis is that in response to the induction of DNA damage, an increase in the dNTP levels is localised specifically to the site of damage, and thus there is no global effect on dNTP pools in the cell that can be quantified with present methods. Although the amount of dNTPs required for many repair events would be low (Niida et al., 2010), the kinetic affinities of repair DNA polymerases for dNTP substrates would require much higher concentrations of dNTPs than those found outside of the S phase in order to catalyse DNA synthesis; thus, compartmentalised production of dNTPs at repair sites might be necessary. Given that RNR is responsible for the production of diphosphates, and notably dUDP and not deoxythymidine diphosphate (dTDP), additional dNTP metabolic enzymes would also be needed at damage sites to produce dNTPs in a balanced composition. With regards to dTTP biosynthesis, thymidylate kinase (TMPK, encoded by *DTYMK*), which produces dTDP from dTMP, has been observed at DNA damage sites (Hu et al., 2012) together with nucleoside diphosphate kinase 3 (NME3) (Tsao et al., 2016), which phosphorylates dNDPs to dNTPs. This recruitment is facilitated by interactions with the histone acetyltransferase TIP60 (also known as KAT5) (Hu et al., 2012; Tsao et al., 2016), which is needed for chromatin remodelling at damaged sites. Cytidylate kinase (CMPK, encoded by *CMPK1*) also forms a complex with TIP60–RNR (Tsao et al., 2015). As a reduction product of RNR is dUDP, which can be phosphorylated to dUTP and mis-incorporated by DNA polymerases, TMPK recruitment, which occurs in an ATM-dependent manner (Hu et al., 2019), is needed to increase the dTTP/dUTP ratio to prevent dUTP incorporation and accumulation of genomic uracil (Hu et al., 2012). Additionally, the dUTP hydrolase dUTPase is also important in preventing accumulation of genomic uracil, particularly in the context of high RRM2 expression (Chen et al., 2016). Highlighting the potential relevance of this in cancer biology, low dUTPase and high RRM2 expression in tumours correlates with poor prognosis in patients with colorectal cancer (Chen et al., 2016). Despite identification of several dNTP biosynthetic enzymes at DNA damage sites in individual studies, a global understanding of this process is still lacking. Several authors have performed proteomic studies at broken forks upon treatment with agents that cause DNA damage (e.g. hydroxyurea and camptothecin) to elucidate the factors recruited to the DNA damage sites (Dungrawala et al., 2015; Nakamura et al., 2021). This provides large amounts of data that could be exploited to understand the association of DNA damage and repair to dNTP metabolism; however, given the emerging roles of nucleotide metabolic enzymes in DNA repair via non-catalytic functions (see Box 2), cautious interpretation of these findings would be needed.

## Consequences of dNTP pool perturbations on genome stability

**Fig. 3.**
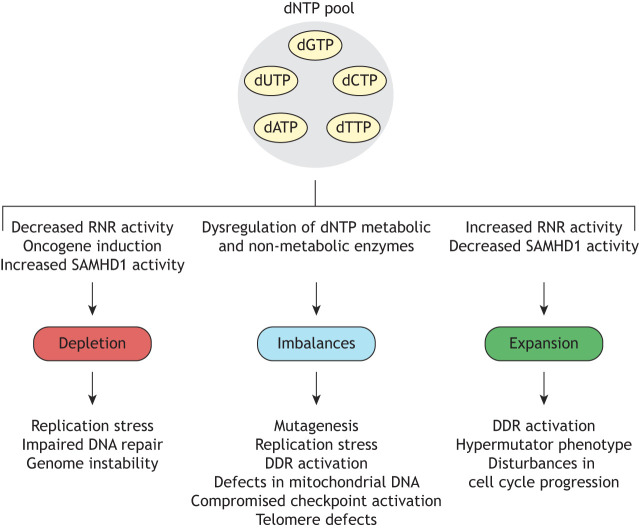
**Dysregulation of dNTP pools impacts genome stability.** Overview of the causes and consequences of deoxynucleoside triphosphate (dNTP) pool perturbations. Many factors can compromise dNTP pool composition, leading to depletion, imbalance or expansion of dNTP pools (note that these are not mutually exclusive, as depleted or expanded pools are often imbalanced). Factors include (dys)regulation of dNTP metabolism by oncogenes and tumour suppressors, together with mutation or altered expression of dNTP and non-dNTP metabolic genes. Subsequently, abnormal dNTP pools can impact genome integrity via multiple mechanisms. These can range from promoting mis-incorporation of dNTPs during DNA synthesis to generate mutations, to more broadly perturbing DNA replication dynamics and cell cycle progression. Here, many open questions remain, particularly regarding the consequences expanded dNTP pools upon genome stability. Abbreviations: dATP, deoxyadenosine triphosphate; dCTP, deoxycytidine triphosphate; DDR, DNA damage response; dGTP, deoxyguanosine triphosphate; dTTP, deoxythymidine triphosphate; dUTP, deoxyuridine triphosphate; RNR, ribonucleotide reductase.

Despite their evident heterogeneity, cancer cells can often harbour common key mutations in oncogenes, tumour suppressors and DNA repair genes that consequently promote genomic instability and the accumulation of further genetic alterations (Martinez-Jimenez et al., 2020; Mehrotra and Mittra, 2020). Indeed, defective maintenance of genomic integrity and the appearance of additional genetic insults enable cancer cells to rapidly adapt and evolve resistance to existing therapies (Ben-David et al., 2019), but this can also be a double-edged sword, as many therapeutic approaches in use exploit these vulnerabilities to (specifically) kill tumour cells. Preservation of dNTP homeostasis is also crucial to avoid other diseases; one of the most studied examples is the interconnection between mitochondrial DNA (mtDNA) depletion and multiple deletions syndromes (MDDS), a group of diseases associated with defects in mtDNA, and mutations in certain nucleotide metabolic enzymes (reviewed by Ramon et al., 2021). Notably, dNTP pool composition can also be perturbed by the presence of non-canonical nucleotides, which we have not discussed here; for details, we direct readers to a previous review (Rudd et al., 2016). Hence, it is imperative to understand the mechanisms underlying genome instability to improve drug treatments and overcome resistance, which can be tightly associated to the accurate maintenance of dNTP pools. Thus, in this section, we aim to provide an overview of the detrimental consequences described so far that arise upon different nucleotide perturbations (Fig. 3), namely, dNTP pool imbalances and both dNTP pool depletion and expansion.

### dNTP pool imbalance

Preservation of genomic integrity requires not only controlled levels of overall dNTP abundance, but also maintenance of relative individual dNTP levels. Imbalances in the dNTP ratios can lead to DNA polymerase errors (i.e. mis-incorporation of dNTPs) and to a hypermutator phenotype (Box 1) that can trigger subsequent replication stress due to disrupted replication fork dynamics, DDR activation and cell cycle arrest (Pai and Kearsey, 2017; Shu et al., 2020). This process is illustrated through dysregulation of a variety of enzymes directly involved in dNTP metabolism. For instance, dTTP exhaustion through TS inhibition, either pharmacological or OIS-mediated, results in mis-incorporation of dUTP during DNA synthesis, which in turn generates repair-induced abasic sites and potentially induces single-strand breaks and DSBs when removed. Thus, impaired fork progression and replication stress is triggered in this situation (Curtin et al., 1991; Mannava et al., 2013; Van Triest et al., 2000). Furthermore, accumulation of dGTP in SAMHD1-deficient bone marrow-derived macrophages upon deoxyguanosine treatment leads to induction of apoptosis (Davenne et al., 2020). Based on this foundation, the authors suggest that use of forodesine – an inhibitor of purine nucleoside phosphorylase (PNP), which degrades deoxyguanosine – in combination with exogenous deoxyguanosine would act synergistically in patients with SAMHD1 deficiency due to an accumulation of cytotoxic dGTP, which could be exploited in the treatment of CLL. These findings were supported by a second study which suggested a broader applicability of PNP inhibitors in SAMHD1-deficient cancers (Abt et al., 2022). The reason why dGTP overload is especially cytotoxic for the cell compared to excess of other nucleotides is still to be discerned, although hypotheses exist. Physiological levels of dGTP appear to be extremely low compared to the levels of the rest of the canonical dNTPs, so small alterations in relative dGTP levels may lead to drastic disturbances in dNTP ratios. Furthermore, as previously mentioned, RNR is allosterically regulated by dNTPs for the selectivity of the NDP to be reduced. Thus, accumulation of dGTP could indirectly restrict *de novo* biosynthesis of dCTP, which would be additionally detrimental for genome integrity (Davenne et al., 2020).

In a similar fashion, it has been demonstrated that imbalances in the dCTP/dTTP ratio negatively affects DNA replication (Zhang et al., 2013). Expansion of dCTP through downregulation of the dCTPase DCTPP1 (which hydrolyses dCTP to dCMP) also triggered dTTP pool depletion and the accumulation of dUTP (Martinez-Arribas et al., 2020). Here, the authors also reported activation of the DDR, increased mutation rates in both nuclear and mitochondrial DNA, and disturbances in cell cycle progression. In HeLa cells, accumulation of high intracellular dCTP levels due to depletion of cytidine deaminase (CDA), the enzyme involved in deamination of (deoxy)cytidine to (deoxy)uridine, negatively affects replication fork progression and gives rise to under-replicated genomic DNA (Gemble et al., 2015). A recent study found that CDA localises to replication forks and that CDA overexpression in pancreatic ductal adenocarcinoma models improves replication dynamics, suggesting that it could be part of a machinery aimed at locally increasing the provision of nucleotides during DNA replication (Lumeau et al., 2024). Indirect perturbation of dNTP pools can also be found by dysregulation of DNA repair, as increased dCTP and dTTP levels were observed in an MMR-deficient yeast model, which led to replication errors in specific DNA sequence motifs due to mis-insertions and defective polymerase proofreading activity (Watt et al., 2016). Recently, it was also found that disturbances in the nucleotide ratios do not prevent the G1/S checkpoint, enabling cell entry into the S phase, during which the resulting replication stress owing to dNTP pool imbalance is sensed by the ATR pathway, allowing prolongation of the S phase until correct dNTP proportions are restored for DNA replication (Diehl et al., 2022).

Imbalances in dNTP pool composition, specifically, enlargement of a single dNTP species within the pool, has also been functionally linked to controlling telomere length. In a recent study, whole-genome CRISPR screening identified a role for thymidine nucleotide metabolism in telomere homeostasis (Mannherz and Agarwal, 2023). Specifically, it was found that loss of dTTP biosynthetic enzymes (TK1 and TS) resulted in telomere shortening, whereas loss of dTTP catabolic enzymes (SAMHD1) resulted in telomere lengthening. A previous study had also shown that expansion of single dNTP species, also controlled by SAMHD1, could regulate telomere length; however, it was not dTTP but rather dGTP (D'Aronco et al., 2023). Potentially, these studies could be reconciled when considering allosteric regulation of RNR, as dTTP promotes the reduction of guanosine diphosphate (GDP) to deoxyguanosine diphosphate (dGDP), and thus expansion of the dTTP pool would promote dGTP biosynthesis. dGTP is known to be rate limiting for telomerase activity (Maine et al., 1999); however, this remains to be investigated further.

### dNTP pool depletion

As dNTPs are the building blocks for DNA synthesis and repair, deficient supply can subsequently lead to inhibition of DNA replication and fork stalling, a process known as replication stress, which may progress to replication fork collapse (Box 1). Accordingly, low dNTP levels also impair DNA repair. Thus, if replication stress is not resolved, it may lead to a hypermutator phenotype and DNA breaks, as well as copy number alterations (Box 1) and chromosomal rearrangements (Box 1) (Primo and Teixeira, 2019). In agreement, it has been reported that, although RRM1 protein levels do not fluctuate through the cell cycle, RRM1 activity is enhanced once phosphorylated at S559 by CDK2/cyclin A during S/G2 phase, which is necessary to sustain DNA replication. Thus, mutations in S559 impair RRM1 phosphorylation and consequently affect replication fork progression, leading to the accumulation of cells in S phase, as well as an increase in DNA damage markers and chromosome and chromatid breaks (Shu et al., 2020). Interestingly, depletion of dNTP pools through hydroxyurea-mediated inhibition of RNR also leads to slower replication dynamics that ultimately trigger replication stress (Poli et al., 2012). Of note, inhibitors of RNR are powerful tools within the DNA repair research community and have been used to characterise the cellular response to replication fork stalling, facilitating the mapping of the proteome surrounding stalled replication machinery, in addition to delineating the many pathways of fork stabilisation and restart (Berti et al., 2020).

Depletion of dNTPs can be associated with oncogene-induced replication stress in cancer development (Kotsantis et al., 2018). On the one hand, oncogene activation triggers uncontrolled hyperproliferation. Under this situation, cells undergo persistent DNA replication and origin firing that can exhaust dNTP levels, leading to fork stalling, replication stress and DSBs, activating the DDR (Graziano and Gonzalo, 2017). Indeed, it has been reported that replication stress produced by overexpression of the cyclin E1 oncogene (*CCNE1*) can be counteracted upon exogenous addition of nucleosides or activation of nucleotide metabolism (Bester et al., 2011). In a similar fashion, inhibition of the WEE1 kinase in mammalian cells results in exacerbated cyclin-dependent kinase (CDK)-dependent firing of replication origins, responsible for dNTP depletion and replication stress (Beck et al., 2012) (Pai et al., 2019). On the other hand, it has also been shown that overexpression of oncogenes directly affects and limits dNTP metabolism. Several groups have reported downregulation of RNR and TK1 in oncogene-expressing cells prior to induction of senescence (Aird et al., 2013; Mannava et al., 2013). Supporting this hypothesis, oncogene-induced replication stress could be prevented by nucleoside supplementation or restoration of RRM2 expression (Aird et al., 2013; Mannava et al., 2013).

### dNTP pool expansion

In stark contrast to the wealth of information available about the consequences on genome stability upon imbalanced and depleted dNTP pools, our knowledge about the impact of dNTP pool expansion on genomic integrity remains limited. One reason could be because expansion of dNTPs usually does not occur in the same proportion for each dNTP, and so, typically, dNTP pool imbalances are observed instead. During the cell cycle, dNTP pools expand to allow efficient genome duplication during the S phase, which is facilitated by the regulation of RNR and SAMHD1. For the expansion of dNTPs outside of the S phase and consequences of the expansion, we mainly find precedents in yeast and bacteria. In these models, dNTP accumulation is facilitated predominantly by upregulation or dysregulation of *de novo* biosynthesis via RNR. Different studies have shown that stimulation of RNR activity, both directly or activated by DNA damage, can lead to an increase in mutations (Davidson et al., 2012; Gon et al., 2011). For example, one study interrogated the link between different expanded and imbalanced dNTP pools and mutagenesis using a panel of RNR-defective yeast models (Schmidt et al., 2019). Here, the strongest mutators had a dNTP imbalance in which three out of the four dNTPs were elevated, especially if dGTP levels were particularly high among the three expanded dNTPs. Furthermore, in the absence of functional DNA replication fidelity mechanisms (i.e. MMR or polymerase proofreading), these imbalances caused growth defects or lethality. The proposed mechanism for induction of these mutations was combinatorial, in which the underrepresented dNTP promotes base substitutions and deletion events, whereas the overrepresented dNTPs promote mismatch extension independent of the templated sequence (Schmidt et al., 2019). An additional explanation is that elevated dNTP levels could promote activity of inaccurate trans-lesion synthesis (TLS) DNA polymerases in addition to reducing the fidelity of replicative polymerases (Lis et al., 2008; Sabouri et al., 2008). TLS polymerases are typically used to replicate lesion-containing DNA templates owing to their inherent flexibility, but as they have lower affinity for dNTP substrates on undamaged templates compared to replicative polymerases, DNA synthesis by these low-fidelity enzymes could potentially be favoured. However, it is worth noting that evidence from bacteria demonstrated that inactivation of TLS polymerases had little effect upon mutation rates associated with dNTP pool expansion (Gon et al., 2011). Additionally, using an RNR-mutant *S. cerevisiae* strain, another study reported that high dNTP concentrations compromised DNA replication due to reduced assembly of the pre-initiation complex, which delayed S phase entry (Chabes and Stillman, 2007). Accordingly, the RNR mutant was synthetic lethal with the *orc2-1* and *orc5-1* origin recognition mutants, characterised by a low number of active origins of DNA replication. The results suggested that unusually high dNTP levels in the G1 phase are detrimental due to defective activation of pre-replication complexes, thus explaining why dNTP levels are so tightly regulated in this cell cycle phase. Besides, contributions in the cancer field show that upregulation of RRM2 leads to higher mutation rates and promotes replication stress and tumorigenesis (Chen et al., 2016; Xu et al., 2008).

As expansion of dNTP pools can also originate from SAMHD1 depletion, different authors have evidenced genomic instability in human cell models that lack SAMHD1. For instance, nucleotide levels appear to influence non-homologous end-joining repair, as both HEK293 CRISPR-engineered SAMHD1 knockout and catalytically dead mutant (K312A) cell lines presented more frequent insertions at chromosomal junctions, suggesting that the phenotype is caused by loss of SAMHD1 catalytic activity (leading to the expansion of dNTPs) and not by SAMHD1-mediated DNA repair (Akimova et al., 2021). Exogenous supplementation of deoxynucleosides further validated these observations. In a SAMHD1-deficient mouse model, lower NTP/dNTP ratios correlated with less ribonucleotide mis-incorporation in mtDNA, although it did not appear to affect mtDNA copy number or integrity (Wanrooij et al., 2020). Furthermore, fibroblasts from individuals with Aicardi-Goutières syndrome, characterised by SAMHD1 deficiency, have expanded dNTP pools coupled with activation of the DDR and genome instability (Kretschmer et al., 2015).

## Exploiting dNTP homeostasis as a cancer target

Targeting DNA metabolism via inhibition of dNTP metabolic enzymes was among the first chemical approaches to successfully treat cancer, an approach that has stood the test of time and one that we revisit within the present paradigm of precision cancer medicine.

### Antimetabolites – altering dNTP pool composition

**Fig. 4.**
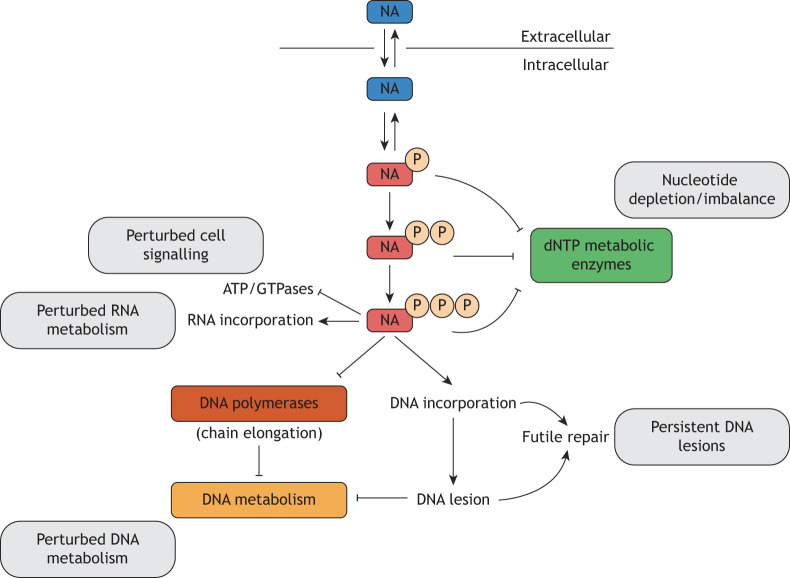
**Overview of the distinct modes of action of nucleoside-based cancer drugs.** Nucleoside analogues (NAs) are intracellularly converted into their monophosphate, diphosphate and triphosphate metabolites by nucleoside and nucleotide kinases. Each metabolite can have a distinct mode of action that also depends upon the chemical modification present on the base or sugar moiety of the particular NA. Phosphorylated metabolites can be potent inhibitors of deoxynucleoside triphosphate (dNTP) biosynthetic enzymes (e.g. thymidylate synthase and ribonucleotide reductase), whereas triphosphate metabolites can be incorporated into nucleic acids by polymerases or perturb activity of ATP and GTPases by mimicking the endogenous nucleotides. Although many NAs are incorporated into DNA, the resulting perturbation on DNA metabolism can differ widely depending on the particular chemical modifications present on the NA. These can range from inhibition of DNA chain elongation to induction of persistent DNA lesions requiring specific DNA repair pathways.

Antimetabolites have long been exploited in cancer treatment. These are small molecules similar in structure to endogenously occurring metabolites, such as nucleosides and folate, and thus alter the composition of the metabolite pool and compete with their endogenous counterparts for use in cellular metabolism. As cancer cells present a constant demand of substrates for DNA synthesis and cell proliferation, they rely on dNTP biosynthesis, in theory making them more susceptible to use of antimetabolites and their subsequent cytotoxic effects (Luengo et al., 2017; Tsesmetzis et al., 2018). The rationale behind this strategy also exploits the intrinsic upregulation of genes involved in dNTP biosynthesis, such as those encoding TK1, RRM2 and TS, among others, which several studies have found to be among the top metabolic enzymes frequently overexpressed in several cancer types (Irwin et al., 2017; Nilsson et al., 2014). In the case of nucleoside analogues, the nucleoside prodrugs are first transported inside the cell where they are metabolically activated by phosphorylation. Induction of genomic instability then occurs through distinct mechanisms, as previously reviewed in detail (Tiwari, 2012; Tsesmetzis et al., 2018) and overviewed in Fig. 4. In general, these compounds either interfere with *de novo* nucleotide metabolism through inhibition of key enzymes, altering the levels of (d)NTPs, or they mis-incorporate into DNA or RNA instead of the canonical nucleotides (Tiwari, 2012; Tsesmetzis et al., 2018). This can result in perturbed nucleic acid metabolism through distinct molecular mechanisms, for instance, inhibition of DNA synthesis (Huang et al., 1990; Rodriguez et al., 2003), induction of DNA–protein crosslink lesions (Carnie et al., 2024) and DNA repair-mediated DNA damage (Swann et al., 1996), together with perturbation of RNA metabolism including ribosome biogenesis and mRNA processing (Chen et al., 2023 preprint; Liang et al., 2022). Altogether, this single class of conventional chemotherapeutic agents encompasses multiple distinct modes of action (Fig. 4) and has repeatably demonstrated its ability to prolong the lives of patients with cancer (Comella et al., 2000; Frasci et al., 2001; Mayer et al., 1994). Accordingly, these agents are widely used in cancer therapy and many are part of the standard-of-care treatments for common malignancies. Despite widespread use, there are drawbacks to these agents, including a narrow therapeutic window and wide interpatient variability in pharmacokinetic parameters (Ciccolini et al., 2016; Tsesmetzis et al., 2018). Therefore, a key area of research is to improve our understanding of the factors controlling the efficacy of these therapies and to exploit this information to rationally refine their clinical use (Ciccolini et al., 2016; Rudd, 2023).

### Indirect targeting via signalling pathways

The interconnection of dNTP metabolism with other cellular pathways, together with regulation by these pathways, can also be exploited for indirect targeting. For example, yes-associated protein (YAP, also known as YAP1) and transcriptional co-activator with PDZ-binding motif (TAZ, also known as tafazzin) are components of the Hippo signalling pathway, which is involved in the regulation of cell proliferation and apoptosis and has been associated with cancer progression and metastasis (Yamaguchi and Taouk, 2020). The expression of the principal enzymes in dNTP biosynthesis, such as RNR, TS and TK1, is regulated by YAP/TAZ. As a result, increased YAP activity may contribute to gemcitabine resistance in breast cancer and overcome RAS-induced senescence (Santinon et al., 2018). The identification of a potent inhibitor against YAP/TAZ, K-975, has already shown increased lifespan in malignant pleural mesothelioma xenograft mice, suggesting that YAP/TAZ targeting could provide another considerable therapeutic option (Kaneda et al., 2020).

Also regulating dNTP biosynthesis is ATR, which, as well as being crucial for the preservation of genomic stability (e.g. by preventing fork collapse during replication stress; Lecona and Fernandez-Capetillo, 2018), also controls the correct supply of dNTPs for DNA replication via regulation of RNR. This can be achieved either by upregulating RNR transcription (Buisson et al., 2015) or by inhibiting its proteasomal degradation (D'Angiolella et al., 2012). Additionally, nucleoside salvage is limited by ATR via regulation of DCK expression (Beyaert et al., 2016; Le et al., 2017; Poczta et al., 2019). Consequently, inhibition of ATR would indirectly deplete dNTP pools and hence limit cancer progression. Although ATR inhibition alone was previously shown to prolong survival in *in vivo* models of B-cell acute lymphoblastic leukaemia, it was insufficient to be therapeutically relevant; however, combined with ATR, RNR and DCK inhibitors, it proved to be an extremely effective strategy against this haematological malignancy (Le et al., 2017). Several groups and pharmaceutical companies have focused on the identification of ATR inhibitors for cancer treatment, and four molecules targeting ATR are currently in clinical studies, namely, VX-970, VX-803, BAY1895344 and AZD6738 (Bradbury et al., 2020). In fact, ATR inhibitors have already shown promising results in combination with nucleoside analogues, supporting the clinical use of ATR inhibitors. For instance, acute myeloid leukaemia cell lines were more sensitive to the RNR inhibitor gemcitabine or hydroxyurea when co-treated with the ATR inhibitor VE-970 (Fordham et al., 2018).

## Conclusions and future perspectives

Homeostasis of dNTP pools is integral to the maintenance of genome stability. Reciprocally, metabolism of the DNA or, more specifically, dysfunctional metabolism can likewise impact the size and composition of dNTP pools. This is a relationship that is fundamental to tumorigenesis. Underscoring this is the knowledge that key oncogenes and tumour suppressors – the architects of cancer biology – all control dNTP metabolism as one means of influencing cell growth and genome plasticity. Furthermore, several studies have ranked enzymes involved in dNTP biosynthesis among the most dysregulated metabolic genes in cancer. Accordingly, dNTP metabolic enzymes and the DNA itself are two anti-cancer targets that have stood the test of time, and with in-depth knowledge of the interplay between these pathways and how different cancer drugs perturb them, we can begin to refine our efforts to target them. For instance, with an understanding of how a particular DNA lesion of a chemotherapeutic agent can disturb dNTP pools, mechanism-based drug combinations can be designed to exploit this imbalance to promote cancer cell killing. Opportunities for refinement also lie in employing new therapeutic modalities to re-target these long-standing pan-cancer dependencies. However, although we have a deep understanding of some dNTP disturbances, in particular, dNTP pool depletion, through small molecules and genetic tools targeting RNR, our understanding of dNTP pool expansion is by comparison severely lacking. Here, efforts should be made to generate novel chemical and genetic tools to artificially induce expansion of dNTP pools, for instance, by targeting SAMHD1, which will allow the research community to interrogate the consequences of this phenomenon upon genome stability and cancer biology, such as the mutational fingerprints of these dNTP pool perturbations. Additionally, use of such tools to delineate the relationship between pro-mutagenic (relevant for malignant transformation) and pro-cytotoxic (relevant for therapeutic efficacy) dNTP pool perturbations would be an important area of future research. Furthermore, new experimental approaches are required to interrogate the existence of local dNTP pools, as this is a major gap in our current understanding. Perhaps these efforts could build upon recently developed methods, such as laser capture microdissection of sub-nuclear locations coupled with highly sensitive methods to measure dNTPs (Breiner et al., 2019), or by employing split fluorescence proteins that have been used to detect non-canonical dNTPs (Fujikawa et al., 2024). Although we have clear evidence of re-localisation of dNTP biosynthesis enzymes to DNA damage sites, it becomes more and more apparent that many of these enzymes have additional non-catalytic functions in the repair of DNA damage. Although information can be gleaned from the use of small-molecule inhibitors or catalytic mutants, the impact of edge-specific genetic (edgetic)-like perturbations, in which a partially functioning protein could have altered biochemical and biophysical interactions with additional functional implications (Zhong et al., 2009), cannot be excluded. Thus, direct measurement of local dNTPs will be key. Altogether, several important knowledge gaps remain in our understanding of the relationship between dNTP metabolism and genome stability, and the development of new technologies and experimental models will no doubt allow the community to answer these questions.
